# Evaluation of Fitting Accuracy of Light- and Auto-Polymerizing Reline Materials Using Three-Dimensional Measurement Techniques

**DOI:** 10.3390/polym17020201

**Published:** 2025-01-15

**Authors:** Miona Utsumi, Natsuko Murakami, Toshiki Yamazaki, Asuka Hirata, Kohei Komine, Bin Li, Kensuke Takakusaki, Junichiro Wada, Noriyuki Wakabayashi

**Affiliations:** Department of Advanced Prosthodontics, Institute of Science Tokyo, Tokyo 113-8510, Japan; utsurpro@tmd.ac.jp (M.U.); t.yamazaki.rpro@tmd.ac.jp (T.Y.); hirata.a.rpro@tmd.ac.jp (A.H.); komine.rpro@tmd.ac.jp (K.K.); namieli.bin@gmail.com (B.L.); k.takakusaki.rpro@tmd.ac.jp (K.T.); wadajun.rpro@tmd.ac.jp (J.W.); wakabayashi.rpro@tmd.ac.jp (N.W.)

**Keywords:** light polymerization, reline, removable partial denture, fitting accuracy

## Abstract

Light-polymerizing reline materials offer improved chairside workability compared to conventional auto-polymerizing reline materials, addressing the partial denture (RPD) incompatibility caused by residual ridge resorption owing to long-term use. This study evaluates the fitting accuracy of relined materials by combining conventional fitting tests with three-dimensional (3D) measurements for detailed analysis. Light-polymerizing reline material (HikariLiner^®^, Tokuyama, Tokyo, Japan, LP) and auto-polymerizing material (Rebase III^®^, Tokuyama, AP) were used. The gaps formed between the relined denture base and the simplified edentulous model were evaluated. The displacement and deviation of the experimentally relined RPDs on the partially edentulous models were analyzed using 3D data superimposition. In the edentulous model, the gaps at all measurement points were significantly smaller for the AP than in the LP. Moreover, the alveolar ridge crest gap was significantly larger than that at other sites. In the partial denture model, the RMS values at the residual ridge crest were significantly lower for the AP. The evaluation method using 3D scanning and comparison was suitable for a detailed fit analysis. Further improvements in the scanning accuracy may enhance future assessments. Therefore, the evaluation method using 3D scanning and comparison was suitable for effectively analyzing the fit of relines, necessitating further accuracy improvements.

## 1. Introduction

Removable partial dentures (RPDs) are an effective solution for restoring esthetics and masticatory function in patients with partial edentulism [1]. However, prolonged use of dentures reduces the fit between the denture tissue surface and the mucosa of the residual ridge owing to ridge resorption. Decreased denture stability can damage the abutment teeth and residual ridges [2,3]. Therefore, relining procedures that enhance the fit are essential for maintaining long-term denture stability [4]. Moreover, direct intraoral relining is a cost- and time-efficient procedure that allows patients to retain their dentures throughout the process, eliminating laboratory intervention [5].

The auto-polymerizing reline material, commonly used as a conventional direct reline material, uses poly (methyl methacrylate) (PMMA) as its base and hardens through a polymerization reaction when the monomer and polymer are mixed [6]. However, their limited working time poses challenges, particularly for RPDs with remaining teeth. If the material enters the undercut areas of the cervical region during intraoral manipulation, it may harden and render the denture irretrievable [6]. In elderly patients with gingival recession or tooth mobility owing to periodontitis [7], improper relining can further increase the risk of damaging the remaining teeth in elderly patients with gingival recession or tooth mobility owing to periodontitis.

However, the light-polymerizing reline material was polymerized outside the oral cavity after the denture was positioned in the mouth. Polymerization begins only upon light irradiation, ensuring that the material maintains a rubber-like state, enabling removal of excess resin, and facilitating denture removal, even from undercut areas [8,9]. These characteristics render light-polymerizing reline materials highly advantageous for chairside procedures, offering excellent workability intraorally and outside the oral cavity.

Previously, the fitting accuracy of denture base materials was evaluated by measuring the gap between the denture and a reference model simulating edentulous conditions [10,11]. However, contact with an undeformable model can lift the denture, making it challenging to assess the actual fit accurately. This method highlights polymerization shrinkage but provides limited information on denture sinking or compression against the mucosa and areas of proper fit.

In partial dentures, the retainers determine the position of the denture. Thus, achieving a proper fit between the denture base and mucosa of the residual ridge is essential after relining. Recently, three-dimensional (3D) measurements using 3D scanners have been employed to examine the fitting accuracy of complete dentures, enabling the quantitative evaluation of the deviation between the denture tissue surface and the mucosa of the residual ridge [12,13]. However, although 3D measurements have been used to evaluate the fit of RPD frameworks [14,15,16], information on the fitting accuracy of RPD bases remains scarce. This study aimed to evaluate the fitting accuracy of relined partial dentures using a 3D measurement method and investigate how the denture tissue surface sinks or lifts relative to the mucosa of the residual ridge after relining.

In this study, (1) the gap between the reference model and the relined denture base was assessed using a simple edentulous model; (2) a 3D comparison was performed on experimental removable partial dentures with metal frameworks to evaluate the impact of different polymerization methods of reline material on the displacement and deviation of the denture tissue surface relative to the mucosa of the residual ridge.

## 2. Materials and Methods

### 2.1. Materials

Two direct relining materials were used in this study: a light-polymerizing reline material (Hikari Liner, Tokuyama, Tokyo, Japan), LP, and an auto-polymerizing reline material (Rebase III, Tokuyama, Tokyo, Japan), AP (Table 1).

### 2.2. Fitting Accuracy Test on the Simple Edentulous Model

#### 2.2.1. Sample Preparation for Fitting Accuracy Test

**Figure 1:**
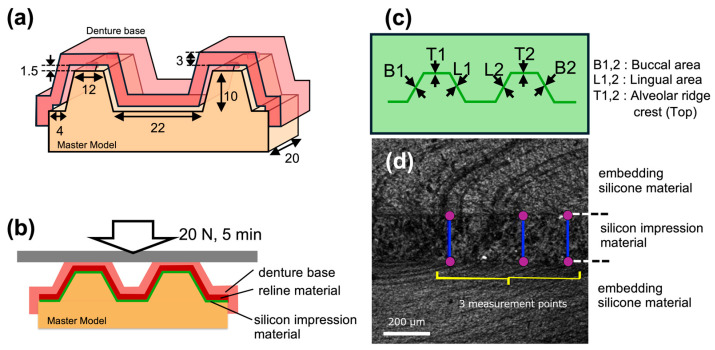
Illustrations of fitting accuracy test using simplified edentulous ridge model. (**a**) Dimensions of the simplified edentulous ridge model (master model) and the experimental denture base. (**b**) During the fitting accuracy test, the gap between the relined denture and the master model was filled with silicone impression material. (**c**) Silicone impression material representing the gap was embedded in silicone resin for further handling, and measurements were taken in each designated area. (**d**) The thickness of the silicone impression material (representing the gap) was measured using a laser microscope (OLS4000, Olympus, Tokyo, Japan).

A simplified brass model (K887-01-000A, Morita, Kyoto, Japan) [17] simulating an edentulous maxillary ridge was replicated using silicone impression material (Duplicone, Shofu, Kyoto, Japan), and a type IV stone master model (New Fujilock, GC, Tokyo, Japan) was prepared. In this master model, denture bases with 1.5 mm spacers (*n* = 10 per material) were fabricated by pouring denture base resin (Procast DSP, GC, Tokyo, Japan) to define the reline space (Figure 1a).

**Figure 2:**
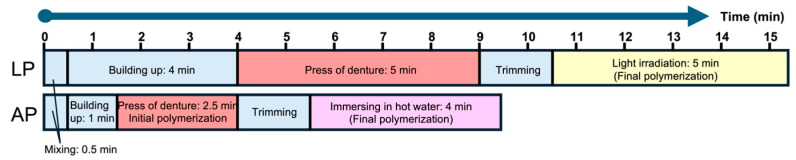
Process diagram ofreline. Relining process of light-polymerizing reline material (LP: upper) and relining process of auto-polymerizing reline material (AP: lower).

Direct reline was performed by a trained operator (U.M.) according to the manufacturer’s instructions for each material (Figure 2). The model was stored in a 37 °C storage cabinet before the study, assuming an intraoral environment. The direct reline materials were mixed at room temperature in a powder–liquid ratio of 2.7 g to 1.7 mL for the LP and 2.9 g to 1.7 mL for the AP. Moreover, they were applied to the denture base and placed on the master model with a 9.8 N load [18]. Denture bases relined with light-polymerizing resin were removed from the model, placed in 60 °C water, and irradiated with light for 5 min. We employed a portable light-curing machine (Portalight, Tokuyama, Tokyo, Japan) with a peak wavelength of approximately 470 nm. Finally, the auto-polymerization reline material was polymerized in 60 °C water for 4 min with a curing accelerator (Tokuso Resin Hardener II, Tokuyama) that was dissolved in it.

#### 2.2.2. Gap Measurement Between Master Model and Relined Denture Base

The fitting accuracy of the relining material was evaluated by measuring the gap between the master model and the tissue surface of the denture base. A silicone impression material (Express 2 Light Body Flow; 3M, Saint Paul, MN, USA) was applied to fill the gap and pressed at a force of 20 N for 5 min (Figure 1b). After the gap was replaced with the silicone impression material, the master model and relined denture base were replaced and embedded in the silicone material (Duplicone, Shofu, Kyoto, Japan) [19]. The measurement points included B1 and B2 in the buccal area, L1 and L2 in the lingual area, and T1 and T2 in the alveolar ridge crest (top). The thickness of the silicone impression material at each measurement point (Figure 1c) was measured thrice by a laser scanning microscope (OLS4000, Olympus, Tokyo, Japan) (Figure 1d).

### 2.3. Three-Dimensional Comparison of Relined RPD

#### 2.3.1. Preparation of Experimental RPD and Relining

**Figure 3:**
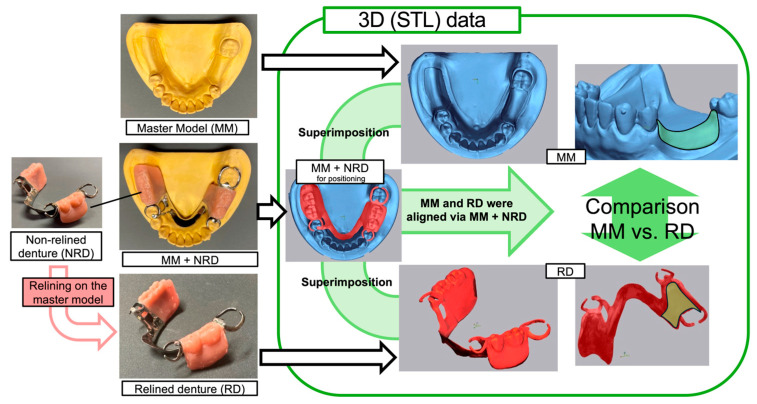
Process of aquation and comparison of 3D data between the residual ridge surface and the tissue surface of relined denture.

To evaluate the 3D relationship between the relined denture tissue surface and residual ridge mucosa, direct relines of experimental RPDs were performed using stone models which represented the pre- and post-resorption conditions (Figure 3).

A non-relined denture (NRD) was placed onto a Master Model (MM) with a resorbed residual ridge, and this assembled model (MM + NRD) was used for positioning. The MM and relined denture (RD) data were aligned by superimposing them onto the MM + NRD, and the residual ridge surface of the MM was compared with the tissue surface of the RD.

A mandibular Kennedy Class II, Modification 1, partially edentulous pre-resorption model (E50-528; Nissin Dental Products, Kyoto, Japan) was used. This model included three prepared abutment teeth (left first premolar #34; left second molar #37; and right second premolar #45). A cobalt–chromium metal framework was fabricated, and a denture base, which included artificial teeth, was created using a silicone core and auto-polymerizing denture base resin (Procast DSP, GC). Three metal frameworks were used for each relining material, yielding nine experimental RPDs per material.

A custom-made post-resorption model (Nissin Dental Products, Kyoto, Japan) was prepared to simulate ridge resorption of approximately 1.5 mm. The relining area was defined, and measurement reference points were embedded for analysis [20]. This resorption model serves as the Master Model (MM) for the relining procedures.

The relining process followed the same conditions and protocols as those used for the simple edentulous model with a measured gap (Figure 2). A relining jig with a plaster core on the occlusal surface was utilized to ensure consistent positioning; the denture was pressed evenly into place in a storage cabinet maintained at 37 °C.

#### 2.3.2. Aquation and Comparison of 3D Data

This study adopted a 3D measurement method from previous studies that compared RPD tissue surfaces with the mucosa of the residual ridge [20]. The master model (MM), non-relined denture (MM + NRD), and relined denture (RD) were scanned using a desktop 3D scanner (EDGE, DOF, Seoul, Republic of Korea) with an accuracy of 7 μm (ISO 12836, Geometrical Product Specifications) manufacturer reported to obtain STL data (Figure 3) [21].

The scanned data were superimposed by 3D measurement software (Geomagic Control X 2020, 3D Systems, Inc., Rock Hill, SC, USA). The MM and RD were aligned through interposed MM + NRD data for positioning. MM was aligned based on residual teeth, whereas RD alignment used artificial teeth and a best-fit algorithm [22].

**Figure 4:**
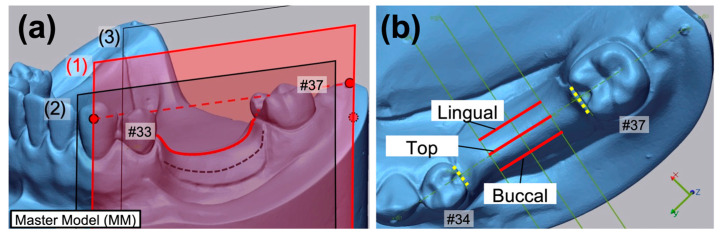
Reference points, planes, and measurement lines. (**a**) Reference plane (1) consisting of three reference points of #33 buccal, #37 distal (solid red points) and posterior wall of model (dotted red point) on the master model, and parallel reference planes (2) and (3); (**b**) measurement lines on the master model separated by the reference planes and the guiding surfaces (yellow dotted line) of #34 and #37.

The 3D deviation between the RD tissue surface and MM mucosa was measured at specific lines on the midline defect on the left side of the MM (Figure 4). The root mean square (RMS) values were calculated to assess fitting accuracy.

### 2.4. Statistical Analysis

To determine the sample size, a power analysis was conducted using G*Power (version 3.1.9.7, Düsseldorf, Germany) to achieve a power of at least 0.8 with α = 0.05.

In both tests, some results of the Kolmogorov–Smirnov test showed normality, whereas Levene’s test did not show equal variances. Therefore, the Kruskal–Wallis test was employed to statistically compare the gap volumes from fitting accuracy tests on edentulous models and the displacements and RMS values from 3D comparisons. Pairwise comparisons were conducted using the Mann–Whitney U test. In addition, the Friedman test was used to evaluate the measurement sites (buccal, maxillary, and lingual) for each test and material. For gap measurements from the fitting accuracy tests that showed significant differences, post hoc tests were conducted for each material. The significance level was set at 5% (α = 0.05). Statistical analyses were performed using the Statistical Package for the Social Sciences (SPSS) software for Windows 11.5J (IBM, NY, USA).

## 3. Results

### 3.1. Gap Thickness in the Fitting Accuracy Test

**Figure 5:**
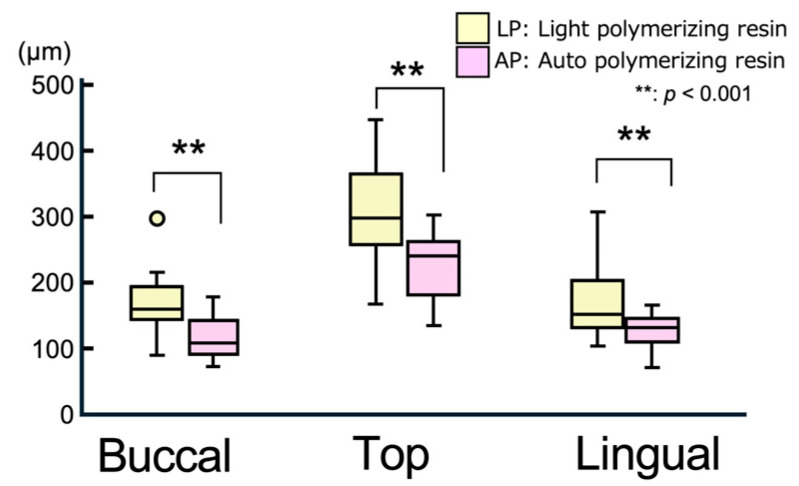
Boxplot of gap thickness at measurement area. Outliers are represented as individual points outside the whiskers.

Figure 5 shows boxplots illustrating the gap thickness between the simplified edentulous ridge model and the relined denture for each material in the buccal, alveolar ridge crest (top), and lingual areas. Table 2 summarizes the mean, standard deviation (SD), median, and interquartile range (IQR) for these measurements. At all locations, the gap for the auto-polymerizing resin was significantly smaller than that for the light-polymerizing resin (*p* < 0.001). Among the three measurement sites, the crest showed significantly larger gaps than other sites (*p* < 0.001), with medians (interquartile ranges) of 301.6 (99.8) μm for the light-polymerizing resin and 247 (75.2) μm for the auto-polymerizing resin. The smallest gaps were observed in the lingual area for the light-polymerizing resin [163.8 (67.8) μm] and in the buccal area for the auto-polymerizing resin [123.7 (41.5) μm].

### 3.2. Displacement and RMS Values in the 3D Comparison

**Figure 6:**
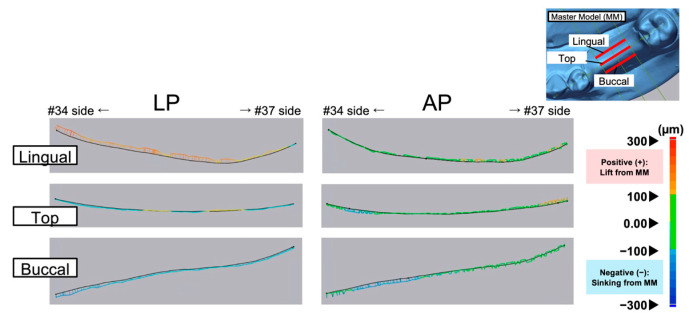
Typical displacement of the tissue surface of the relined denture relative to the master model (MM) surface as the reference (black line).

**Figure 7:**
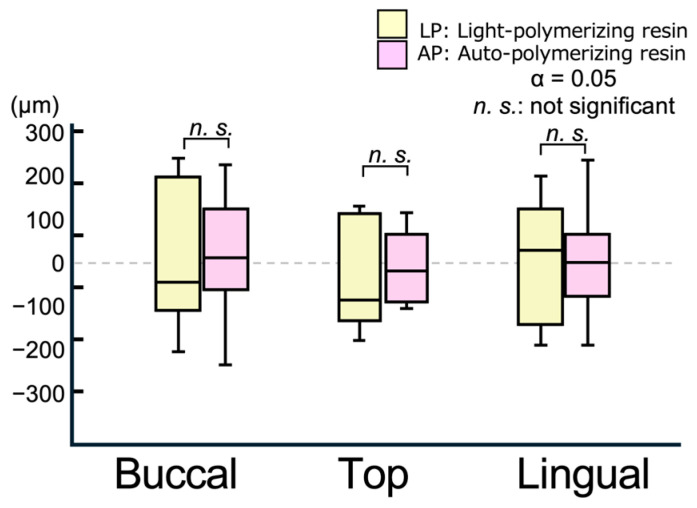
Boxplot of displacement values on the measurement line in a 3D comparison.

**Figure 8:**
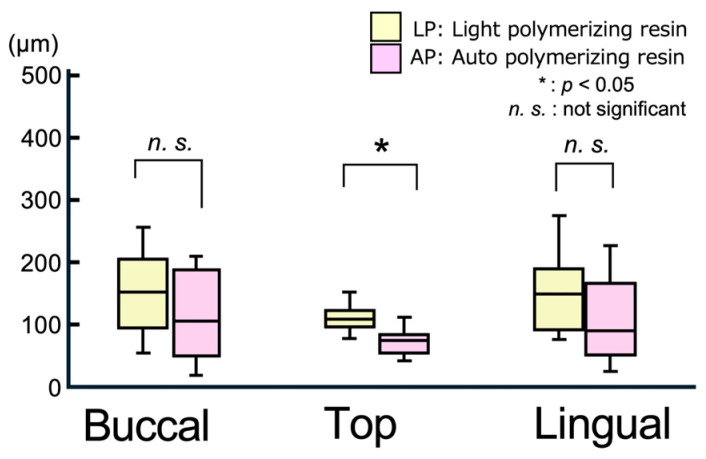
Boxplot of RMS values on the measurement line in a 3D comparison.

Figure 6 illustrates representative displacement results of the relined denture (RD) tissue surface in the normal direction from the measurement line of the master model (MM). Positive values indicate a lifting of the RD from the MM, whereas negative values indicate a sinking of the RD into the MM. Figure 7 and Figure 8 present boxplots showing the displacement values and RMS values, respectively. Table 3 summarizes the mean, SD, median, and IQR for the data shown in Figure 7 and Figure 8.

In all areas, the degree of lift and sinking (Figure 7) of the tissue surface of the relined denture differed relative to the master model, with no significant differences between the materials (Figure 6). Figure 8 shows the RMS values, which indicate the degree of deviation from the master model. The smallest RMS values for both materials were observed at the alveolar ridge crest, with the APs showing a significantly smaller RMS value [90.5 (25.0) μm] than the light-polymerizing resin [123.3 (16.4) μm] at the crest (*p* = 0.015). However, no significant differences were observed between the materials on the buccal and lingual sides. Furthermore, no significant differences were observed in the displacement and RMS values of each material, even when the measurement sites were different.

## 4. Discussion

A fitting accuracy test of the edentulous model was used to evaluate the gaps formed when the relined denture was returned to the master model. Owing to polymerization shrinkage and deformation that took place during the polymerization process of the relining material, the buccal and lingual surfaces made minimal contact, yielding a significant gap at the residual ridge crest (top region). Therefore, while the cumulative vertical lift after polymerization can be easily detected and compared between materials, horizontal deviations caused by pressing techniques during fitting evaluation can introduce significant measurement errors. In this study, the gap in the AP at the crest area was smaller than that in the LP, owing to reduced deformation during polymerization. Wada et al. [17] reported a gap of approximately 200 μm at the ridge crest for heat-polymerizing denture base resin using the same mold. Compared to the gaps observed in this study for LP (301.6 μm) and AP (247 μm), the heat-polymerized resin yielded superior fitting accuracy. This trend aligns with previous findings; for example, [18] demonstrated that heat-polymerized resins generally exhibit smaller gaps than other relining materials.

Compared to the elasticity-free model used in the experiments, the actual mucosa was elastic. Therefore, it is rare for a denture to be lifted and not return to its proper position as defined by the retainer after relining. Thus, 3D data were superimposed to eliminate the influence of other areas and to evaluate the fitting accuracy between the mucosal surface and the relined denture. The displacement values from the reference model indicated that the negative values corresponded to sinking into (or compressing) the ridged mucosa. However, no consistent trends were detected for specific areas in this study. However, the RMS values, representing the absolute value of the displacements, revealed that AP (90.5 μm) showed significantly smaller values than LP (123.3 μm) at the ridge crest (top region). This result is consistent with the gap observed in the edentulous model, demonstrating the superior fitting accuracy of the AP resin.

The 3D comparison results were attributable to various factors, including polymerization methods, errors during the relining process, and inaccuracies throughout the 3D scanning and data comparison process. In this study, no scanning powder was used, allowing the surfaces of the artificial teeth and denture bases to be scanned directly. However, errors may occur during data reconstruction from multiple angles in the software, particularly in the buccolingual direction.

The fitting accuracy was attributable to various factors. For the AP resin, polymerization began immediately upon mixing, enabling prolonged contact with the model during shaping and enhancing fitting accuracy. However, the LP material did not start to polymerize until light curing was initiated, yielding free shrinkage while it was being removed from the model [23]. This yielded significant deformation. The fitting accuracy was attributable to various factors, such as residual stress during removal from the model [23], viscoelasticity of the resin [9], and morphology of the ridge [11,24].

Although regional differences were not prominent in this study, the gap and RMS values at the ridge crest were consistent with those reported previously, supporting the reliability of the fitting accuracy assessment. Future advancements in scanning and superimposition accuracy will enable more precise evaluations.

This study had various limitations. In oral environments, factors such as saliva, temperature changes, and mucosal elasticity affected the fitting accuracy [25]. Aspects of the relining process, such as the powder–liquid ratio, procedure timing, resin flowability, and degree of applied pressure, could affect the outcomes [26]. The fitting accuracy was evaluated on the day the relining was performed. LP resins are known for their excellent dimensional stability, exhibiting minimal changes over extended periods [18]. However, the AP resin was affected by ongoing polymerization and water absorption within 24 h to 1 w after relining [27]. The polymerization rate of these materials affects the degree of residual stress and shrinkage.

In this study, the degree of polymerization and glass transition temperature [28,29] were not measured. However, investigating differences in polymerization methods arising from the choice of polymerization initiators could clarify the relationship between the degree of polymerization and residual stress using techniques such as differential scanning calorimetry (DSC) [30]. This could contribute to more effective evaluations of relining materials and the development of new materials. Furthermore, future studies should prioritize evaluating the long-term fitting accuracy under actual oral conditions.

## 5. Conclusions

Despite the limitations of this study, the AP demonstrated significantly smaller gap and RMS values at the ridge crest than the LP. Therefore, the AP may offer a higher fitting accuracy owing to less deformation occurring during the polymerization process. However, the fitting accuracy of LP was superior to that of the PMMA resin commonly used in new denture fabrication, indicating that LP is sufficiently practical for clinical relining procedures.

The evaluation method using 3D scanning and comparison was suitable for analyzing the detailed fit between the relined dentures and the ridge model. However, the potential for errors in the buccolingual direction highlights the need for further advancements in scanning and alignment accuracies.

This study was based on model evaluation. Therefore, future research must assess the fitting accuracy under oral conditions and determine how these factors influence long-term performance.

## Figures and Tables

**Table 1:** Materials and composition: composition as provided by the manufacturer.

Material	Code	Product and Manufacturer	Powder	Liquid
Light-polymerizing reline material	LP	Hikari-liner, Tokuyama	Polyethyl methacrylate, camphorquinone, coloring material, etc.	Methacrylate monomers, dimethacrylate monomers, etc.
Auto-polymerizing reline material	AP	Rebase III, To-kuyama	Polyethyl methacrylate, benzoyl peroxide, etc.	Methacrylate monomers, dimethacrylate monomers, etc.

**Table 2:** Gap thickness measurements (mean, SD, median, and IQR) for each material in buccal, alveolar ridge crest (top), and lingual areas (unit: μm).

Measurement Area	Material	Mean	SD	Median	IQR
Buccal	LP	179.8	43.1	172.1	46.8
	AP	131.9	29.0	123.7	41.5
Top	LP	311.7	67.9	301.7	99.8
	AP	232.6	45.6	247.3	75.2
Lingual	LP	182.3	54.0	163.8	67.7
	AP	140.3	23.4	145.4	31.4

**Table 3:** Summary of mean, SD, median, and IQR for displacement and RMS values (unit: μm).

Measurement Area	Material	Displacement				RMS			
		Mean	SD	Median	IQR	Mean	SD	Median	IQR
Buccal	LP	15.6	150.0	−36.0	238.2	164.1	58.7	163.3	85.1
	AP	20.9	125.5	16.4	146.1	124.2	65.4	119.7	108.6
Top	LP	−24.6	113.7	−75.3	213.7	123.5	20.4	123.3	16.4
	AP	−4.3	76.7	−13.5	124.0	87.5	19.2	90.5	25.0
Lingual	LP	10.5	131.6	32.2	200.1	160.3	57.9	160.2	84.9
	AP	7.6	109.7	5.4	84.1	119.3	60.8	105.3	93.2

## Data Availability

The data presented in this study are available upon reasonable request from the corresponding author.
